# The role of potassium channels in the mechanism of vasodilatation of human umbilical vein induced by resveratrol

**DOI:** 10.1186/2050-6511-13-S1-A29

**Published:** 2012-09-17

**Authors:** Dragana D Protić, Radmila B Novaković, Svetlana Spremović-Radjenović, Nebojša V Radunović, Helmut Heinle, Vladimir I Kanjuh, Ljiljana C Gojković-Bukarica

**Affiliations:** 1 Institute of Pharmacology, Clinical Pharmacology and Toxicology, Medical Faculty, University of Belgrade, Serbia; 2 Institute of Gynecology and Obstetrics, Clinical Center of Serbia, 11129 Belgrade, Serbia; 3 Institute of Physiology, University of Tübingen, 72076 Tübingen, Germany; 4 Serbian Academy of Sciences and Arts, 11129 Belgrade, Serbia

## Background

Resveratrol (RSV) is polyphenol present in various kinds of food which we consume on daily basis. In the last ten years there has been growing importance of RSV in the literature. It is well known that RSV has many different beneficial effects on human health. RSV is partly responsible for the cardiovascular benefits of red wine. However, the mechanism of vasodilatation induced by RSV is unclear. There are many target molecules of RSV which could play an important role in the mechanism of action of RSV. The aim of our study was to define the role of K^+^ channels in the RSV-induced vasodilatation of human umbilical vein (HUV) denuded of endothelium.

## Methods

HUV rings were precontracted with serotonin (5-HT) or with 100 mM K^+^. Concentration-response curves were obtained by adding increasing concentrations of RSV, from 1 to 100 µM. In order to test the role of vascular K^+^ channels in this vasorelaxation, various K^+^ channels blockers were added to the organ bath 20 minutes before RSV.

## Results

RSV induced concentration-dependent vasodilatation (EC_50_ = 16.5 µM). A selective blocker of ATP-sensitive K^+^ channels, glibenclamide (10 mM), induced a significant shift to the right (p < 0.05) of the concentration-response curve for RSV (EC_50_ = 38.0 µM). 4-Aminopyridine (4-AP, 1 mM), a blocker of voltage-gated K^+^ channels, also induced a significant shift to the right (EC_50_ = 49.0 µM, p < 0.05). Tetraethylamonium (TEA, 10 mM), which predominantly inhibits large conductance Ca^2+^-activated K^+^ channels, and barium chloride (BaCl_2_, 1 mM), which blocks inward rectifier K^+^ channels, antagonized the response to RSV (EC_50_ = 28.0 µM, p < 0.05 and EC_50_ = 50 µM, p < 0.05, respectively). Concentrations of RSV above 10 µM relaxed HUV rings bathed in a medium containing 100 mM K^+^ (EC_50_ = 47 µM, p < 0.05).

## Conclusions

These results suggest that RSV induces endothelium-independent vasorelaxation of HUV. K^+^ channels are involved in the vasodilatation of HUV induced by RSV, when RSV is applied in concentrations up to 10 µM. However, it seems that RSV has an additional, K^+^ channel-independent mechanism of action when applied in concentrations higher than 10 µM.

